# *Diaphorina citri* Induces Huanglongbing-Infected Citrus Plant Volatiles to Repel and Reduce the Performance of *Propylaea japonica*

**DOI:** 10.3389/fpls.2016.01969

**Published:** 2016-12-26

**Authors:** Yongwen Lin, Sheng Lin, Komivi S. Akutse, Mubasher Hussain, Liande Wang

**Affiliations:** ^1^State Key Laboratory of Ecological Pest Control for Fujian and Taiwan Crops, Fujian Agriculture and Forestry UniversityFuzhou, China; ^2^Plant Protection College, Fujian Agriculture and Forestry UniversityFuzhou, China; ^3^Key Laboratory of Biopesticide and Chemical Biology, Ministry of EducationFuzhou, China; ^4^Key Laboratory of Integrated Pest Management for Fujian-Taiwan Crops, Ministry of AgricultureChina, Fuzhou, China; ^5^Institute of Applied Ecology, Fujian Agriculture and Forestry UniversityFuzhou, China

**Keywords:** ladybird beetle, volatiles, huanglongbing disease, insect vector, D-limonene, methyl salicylate

## Abstract

Transmission of plant pathogens through insect vectors is a complex biological process involving interactions between the host plants, insects, and pathogens. Simultaneous impact of the insect damage and pathogenic bacteria in infected host plants induce volatiles that modify not only the behavior of its insect vector but also of their natural enemies, such as parasitoid wasps. Therefore, it is essential to understand how insects such as the predator ladybird beetle responds to volatiles emitted from a host plant and how the disease transmission alters the interactions between predators, vector, pathogens, and plants. In this study, we investigated the response of *Propylaea japonica* to volatiles from citrus plants damaged by *Diaphorina citri* and *Candidatus Liberibacter asiaticus* through olfactometer bioassays. Synthetic chemical blends were also used to determine the active compounds in the plant volatile. The results showed that volatiles emitted by healthy plants attracted more *P. japonica* than other treatments, due to the presence of high quantities of D-limonene and beta-ocimene, and the lack of methyl salicylate. When using synthetic chemicals in the olfactory tests, we found that D-limonene attracted *P. japonica* while methyl salicylate repelled the predator. However, beta-ocimene attracted the insects at lower concentrations but repelled them at higher concentrations. These results indicate that *P. japonica* could not efficiently search for its host by using volatile cues emitted from psyllids- and Las bacteria-infected citrus plants.

## Introduction

Plants are constantly damaged by sap-feeding insects that not only create physical damage while probing but also transmit pathogens that may cause serious disease in plants. Insect-borne pathogens can induce changes in the traits of their primary hosts as well as their vectors that eventually affect the frequency and nature of interactions between hosts and vectors (Eigenbrode et al., 2002; Purcell, 2003). Simultaneous damage by insects as well as pathogenic bacteria induces infested host plants to secrete volatiles that modify not only the behavior of its insect vector but also their natural enemies.

Infected plants have been reported to attract insects infested with pathogens more than a healthy plant (Mann et al., 2009). For example, cucumber mosaic virus (CMV) infected plants emit some chemicals that can manipulate the behavior (increase the attractiveness) of the vector aphids, *Myzus persicae* (Sulzer) and *Aphis gossypii* Glover (Hemiptera: Aphididae) (Mauck et al., 2010). In another study, the phytopathogenic bacterium, *Candidatus* Liberibacter asiaticus (Rhizobiales: Rhizobiaceae) (Las) infected citrus was found to alter the release of specific headspace volatiles and attract more *Diaphorina citri* Kuwayama (Hemiptera: Psyllidae) vectors (Mann et al., 2012). In addition, Las has the ability to manipulate the propensity of psyllids movement and consequently satisfy the vector to promote their spread (Martini et al., 2015). However, de Oliveira et al. (2014) found that the bird cherry-oat aphid, *Rhopalosiphum padi* (Linnaeus) (Hemiptera: Aphididae), which is a vector of cereal yellow dwarf virus (CYDV), a pathogen of wheat, became more vulnerable to attacks by the parasitoid wasp, *Aphidius colemani* Viereck (Hymenoptera: Braconidae) when carrying CYDV. This indicates that plant pathogens alter the metabolites of infected host plants (Rogers and Bates, 2007; Mauck et al., 2010; Van Den Abbeele et al., 2010; Davis et al., 2012; Mann et al., 2012).

Studies have shown that parasitoids are more sensitive to volatiles emitted by infected plants. To attract more insect vectors, Las induces citrus plants to emit more methyl salicylate (Mann et al., 2012). However, simultaneous attack of citrus plants by Las and psyllids, change both the profile and quantity of volatiles secreted (Hijaz and Killiny, 2016). Interestingly, methyl salicylate, produced by Las-infested host plants, is not only used by the bacterial pathogen to manipulate the behavior of its insect vector to promote its own proliferation, but also used by an effective ectoparasitoid of *D. citri*, *Tamarixia radiata* (Waterston) (Hymenoptera: Eulophidae), as a cue to trace its host insect (Martini et al., 2014).

As a consequence, plant pathogens not only regulate the metabolism of host plants, but also influence their vectors, while parasitoids become more sensitive to new metabolites. Polyphagous insect predators are also attracted by herbivore-induced-plant volatiles (Ninkovic et al., 2001; Gencer et al., 2009). It is known that parasitoids are more sensitive to and are attracted by the induced volatiles than their predators (Li et al., 2014). However, little is known about the mechanism that regulates this difference. Therefore, in this study, we used ladybird beetles as models to address the question how ladybird beetles respond to volatiles emitted from host plants that are damaged by insect vectors as well as are infected by plant pathogens.

Las is a phytopathogenic bacterium that is spread by an insect vector, the Asian citrus psyllid, *D. citri*. It is a gram-negative, fastidious, phloem-limited bacterium that causes huanglongbing (HLB) disease in citrus (Roossinck, 2011). Ladybird beetles are known as one of the polyphagous insect predators of *D. citri* in citrus orchards (Michaud et al., 2004). In this study, we investigated the response of a common insect vector *Propylaea japonica* (Thunberg) (Coleoptera: Coccinellidae), in the crop systems of China, to volatiles from citrus damaged by both *D. citri* and Las bacteria. Synthetic chemicals were also used to determine the active compounds in plant volatile blends that affect *P. japonica*.

## Materials and Methods

### Plants

Sour orange (*Citrus aurantium* L.) seeds were cultured in an insect-proof greenhouse at 28°C, 40% RH and L16: D8 for 4 months. The 4-month-old seedlings were infested by grafting them with four pieces of bud wood sticks from a PCR-positive HLB source. The infection was determined and confirmed using PCR as described by Tatineni et al. (2008). Infected (4 month post inoculation) and uninfected 8-month-old citrus plants were used in this study.

### Insect Colonies

*Propylaea japonica* adults and larvae were collected from the fields of Fuzhou, Fujian, China (26.08 N, 119.28 W) in 2014, and reared on *D. citri* that were maintained on *Murraya paniculata* L. (Jack). Five-day-old adult *P. japonica*, which emerged from the established colony (third generation) were selected for experiments. *Diaphorina citri* used for rearing the ladybird beetles and for the experiments were obtained from a colony established in 2014 from field populations in Ganzhou, Jiangxi, China (25.85 N, 114.92 W); and 2- to 3-day-old adults were selected for the tests. This *D. citri* culture was maintained on uninfected sour orange plants in a clean 50 cm × 50 cm × 50 cm netted cage in an insect-proof greenhouse maintained at 28°C, 40% RH, and L16: D8. Psyllids reared on the infested citrus plants were sampled to assess Las bacteria infection by PCR, and the results were positive.

### Olfactometer Assays of *Propylaea japonica*

**FIGURE 1:**
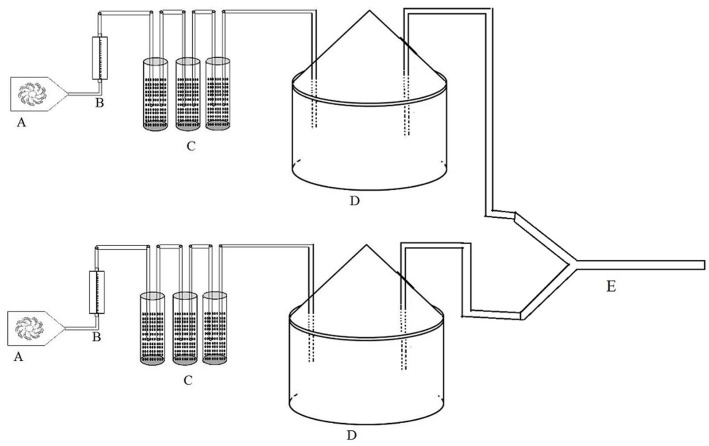
**Schematic diagram of headspace collecting system**. (A) pump, (B) flow meter, (C) air-purify cup, (D) glass jar, and (E) Y-tube.

The response of *P. japonica* to odor sources was studied in two-choice tests with a closed system Y-tube olfactometer, using the method described by Gencer et al. (2009) with a slight modification. Five treatment combinations were used to test the attraction of the chemicals to *P. japonica* with an olfactometer: (1) healthy citrus (HC) vs. Las-infected citrus (LC); (2) healthy citrus infested with psyllids *D. citri* (HCfP) vs. Las-infected citrus infested with psyllids (LCfP); (3) HC vs. HCfP; (4) LC vs. LCfP; and (5) psyllids only vs. fresh air only. For the feeding damage treatments or citrus infestation with the psyllids, 50 adult male and 50 adult female psyllids were placed on plants for 24 h before assays (Mann et al., 2012; Martini et al., 2014). This duration of feeding damage has been proven sufficient to release herbivore-induced volatiles from citrus plants (Mann et al., 2012). Plant samples were placed in 35 cm tall × 25 cm wide dome-shaped volatile collection chambers as described by Lin et al. (2016). For the olfactometer experiment, purified air at the rate of 300 ml min^-1^ was blown into the chambers from the inlet, and then along with headspace and flowed out of the outlet, then went into the arm of the Y-tube (**Figure 1**). A cotton ball was placed at the end of each arm of the Y-tube to block the released ladybird beetle. Adult *P. japonica* were released individually at the entry of the Y-tube and were allowed 300 s to exhibit a behavioral response (Martini et al., 2015). Fifty adult *P. japonica* (male:female ratio 1:1) were individually released into the Y-tubes for each of the five treatments as defined above. Y-tubes were cleaned with hot water (above 60°C) after every set of five insects was tested. Different sets of plant treatments were used to test 50 adult *P. japonica*, and the selected adult ladybird beetles were starved for 24 h before testing. In total, there were five replicates in this experiment.

### Headspace Collection and Volatiles Analysis

Headspace was used to collect volatiles for 24 h with a collecting system described by Pineda et al. (2013). Four treatments were used as described above; HC, LC, HCfP, and LCfP. For the feeding damage treatments, 50 adult male and 50 adult female psyllids were placed on plants for 24 h prior to the bioassays (Mann et al., 2012; Martini et al., 2014). All the treatment plants were placed in clean glass jars inside the collecting chamber as mentioned above. Fresh air was blown into the jar at the rate of 300 ml min^-1^ and flowed out of the headspace through a glass tube filled with absorbent, 200 mg Tenax TA (60/80 mesh; Grace-Alltech, Deerfield, MA, USA). After 24 h collection, the herbivore-induced volatiles, Las-induced volatiles and herbivore-Las-induced volatiles (HBIPVs), which were trapped by Tenax TA, were immediately eluted into 1 ml methenyl trichloride and kept at -20°C until analysis.

The herbivore-induced volatiles, Las-induced volatiles and HBIPVs were analyzed with a gas chromatograph (Agilent Technologies 7890B GC System)-mass spectrometer (Agilent Technologies 5977A MSD) (GC-MS) aligned with HP-5 column (30 mm × 0.25 mm, i.d., 1.0 μm film thickness, Agilent). The GC-MS oven temperature was programmed from 40°C (5-min hold) to 280°C at the rate of 10°C min^-1^. The column eﬄuent was ionized by electron impact ionization at 70 eV. Mass spectra were acquired by scanning from 35 to 350 *m/z* with a scan rate of 5.38 scans s^-1^.

Compounds were identified by using the deconvolution software AMDIS (version 2.64, NIST, USA) in combination with NIST 05 and Wiley seventh edition spectral libraries and by comparing their retention indices with those from the literature. Additional experiments were conducted prior to the bioassays and the results are compiled in the Supplementary documents (Supplementary Experiments 1, 2, and 3 dataset).

### *Propylaea japonica* Response to Synthetic HBIPVs Source

As shown in Supplementary Table 3, D-limonene, methyl salicylate and beta-ocimene volatile compounds collected from the headspace have a significant correlation with the olfactory response of *P. japonica* and therefore were considered for single and blends bioassays. For the experiments, the synthetic version of the three chemicals mentioned above (methyl salicylate, beta-ocimene, and D-limonene) were purchased from Macklin Biochemical Co. Ltd. (Shanghai, China) with approximately 97–99% purity.

For testing single dosage, D-limonene was dissolved in triethyl citrate (TEC) to 50, 25, 17, 10, 1, and 0.1 nmol ml^-1^; methyl salicylate was dissolved in TEC to 0.03, 0.06, 0.15, 0.18, 0.32, and 0.64 nmol ml^-1^; and beta-ocimene was dissolved in TEC to 0.04, 0.08, 0.20, 0.45, 1.00, and 2.00 nmol ml^-1^ concentrations. The range of dilutions used for the bioassays was based on the Supplementary Experiment 2 mentioned above (Supplementary Tables 4 and 5). However, for testing blends, these three chemical compounds were mixed in TEC at different concentrations as follows: (1) blend HC: D-limonene 24.75 nmol ml^-1^ and beta-ocimene 1.00 nmol ml^-1^; (2) blend LC: D-limonene 17.32 nmol ml^-1^, methyl salicylate 0.06 nmol ml^-1^ and beta-ocimene 0.45 nmol ml^-1^; (3) blend HCfP: D-limonene 10.13 nmol ml^-1^, methyl salicylate 0.15 nmol ml^-1^, and beta-ocimene 0.20 nmol ml^-1^; and (4) blend LCfP: D-limonene 1.06 nmol ml^-1^, methyl salicylate 0.18 nmol ml^-1^, and beta-ocimene 0.08 nmol ml^-1^ (Supplementary Table 5).

Then, 1 ml of each suspension solution was applied to a small cotton roll and placed in the collecting chamber. Purified air at the rate of 300 ml min^-1^ was pumped/blown into chambers from the inlet, and then along with volatile and flowed out from the outlet, through the arm of the Y-tube. At the same time, fresh air was forced/pumped directly into the other arm of the Y-tube with a pump at the rate of 300 ml min^-1^ (**Figure 1**). Adult *P. japonica* were released individually at the entry of the Y-tube and allowed 300 s to exhibit a behavioral response as described above (Martini et al., 2015). Y-tubes were cleaned with hot water (above 60°C) after every set of five insects was tested. Different sets of blends (blend HC, blend LC, blend HCfP, and blend LCfP) and single dosages as defined above were used to test 50 adult *P. japonica*, and selected adult ladybird beetles were starved for 24 h prior to the bioassay test, and the experiment was conducted five times.

### Data Analysis

For the Y-tube olfactometer assays, we performed chi-squared tests on the mean values of five replicates. In the Supplementary Experiment, we performed Pearson Correlation to determine the correlation between the olfactory responses of *P. japonica* to citrus volatile blends. We used SPSS 21.0 for overall statistical analyses.

## Results

### Olfactometer Assays of *P. japonica*

**FIGURE 2:**
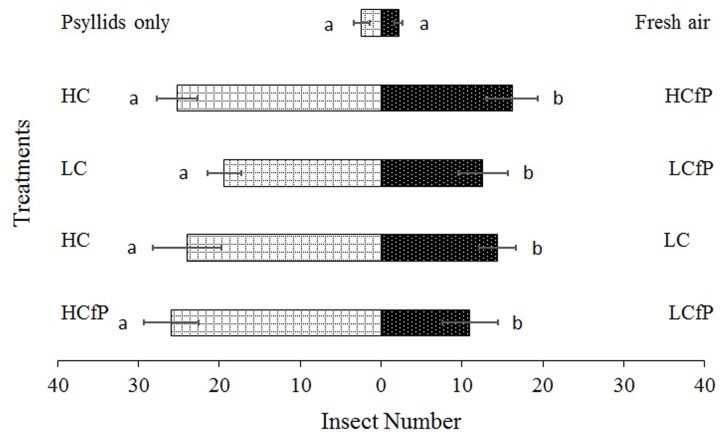
**Olfactometer assays of *Propylaea japonica* with volatiles emitted from treated citrus plants**. Bars indicate mean ± *SE*; means within rows followed by different alphabets indicate significant differences at *P* < 0.05. HC, healthy citrus; LC, Las-infected citrus; HCfP, healthy citrus infested with psyllids; LCfP, Las-infected citrus infested with psyllids.

The results obtained from the olfactometer assays that tested the behavior of *P. japonica* to the different volatiles emitted from damaged citrus plants are shown in **Figure 2** with different effects on the predator. HC treatment was more attractive to *P. japonica* than treatments LC (χ^2^ = 12.89, *df* = 4, *P* = 0.024) and HCfP (χ^2^ = 11.63, *df* = 4, *P* = 0.040). Conversely, treatment LCfP was less attractive to *P. japonica* than treatments LC (χ^2^ = 9.85, *df* = 4, *P* = 0.043) and HCfP (χ^2^ = 17.31, *df* = 4, *P* = 0.004) (**Figure 2**).

### Headspace Collected Volatiles and Analysis

The various volatiles collected from the headspace system from the damaged and infested citrus plants were detected and quantified by GC-MS. Three major chemical compounds (D-limonene, methyl salicylate, and beta-Ocimene) were identified as shown in **Table 1**. All three compounds were found in the various treatments in variable quantities, except methyl salicylate, which was absent in the HC treatment (**Table 1**). There was significant difference (*F*_2,__2_ = 65.01, *P* = 0.002) in the emitted quantity of D-limonene between the treatments. The amounts of D-limonene in treatments HC (13.45 ± 0.99) and LC (10 ± 0.62) did not vary but were considerably higher than in treatments HCfP (6.26 ± 0.73) and LCfP (0.70 ± 0.03) (**Table 1**). Similarly, there was significant difference (*F*_2,__2_ = 165.86, *P* = 0.0001) between the treatments in the quantities of methyl salicylate produced, which was higher in LCfP (0.097 ± 0.006) compared to HCfP (0.055 ± 0.001) and LC (0.028 ± 0.001). In addition, significantly high amounts of beta-ocimene was obtained in HC (0.62 ± 0.04) compared to other treatments (*F*_2,__2_ = 58.84, *P* = 0.002); while the lowest quantity was emitted in LCfP (0.051 ± 0.037) (**Table 1**).

**Table 1:** Quantities (mean ±*SE*) of major compounds from the headspace of treated citrus plants.

Treatment	Major compounds (ng)		
	D-Limonene	Methyl salicylate	beta-Ocimene
HC	13.45 ± 0.99a	ND	0.62 ± 0.04a
LC	10.00 ± 0.62a	0.028 ± 0.001a	0.28 ± 0.03b
HCfP	6.26 ± 0.73b	0.055 ± 0.001b	0.12 ± 0.01c
LCfP	0.70 ± 0.03c	0.097 ± 0.006c	0.051 ± 0.037c

*HC, healthy citrus; LC, Las-infected citrus; HCfP, healthy citrus infested with psyllids; LCfP, Las-infected citrus infested with psyllids; and ND, no detected compound. Means followed by different alphabets within a column represent significant differences at *P* < 0.05*.

### *Propylaea japonica* Responses to Synthetic Compounds

Three compounds, D-limonene, methyl salicylate and beta-ocimene, among the emitted volatiles correlated significantly with the olfactory responses of *P. japonica*, while the remaining were not (Supplementary Table 3). Therefore, D-limonene, beta-ocimene and methyl salicylate were chosen for the bioassays to further study the responses of *P. japonica.*

**FIGURE 3:**
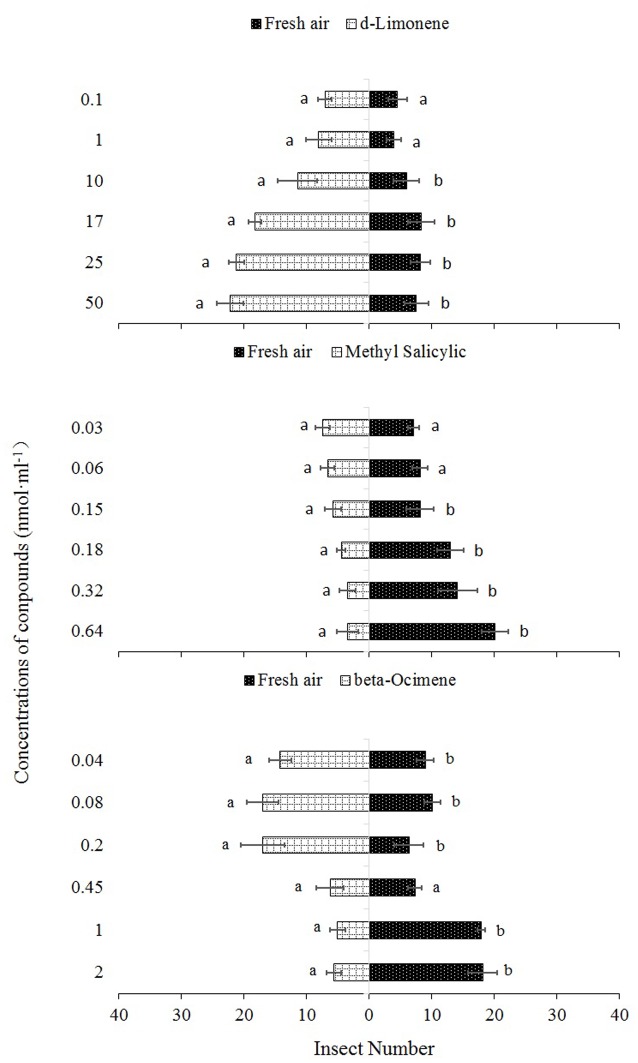
***Propylaea japonica* responses to three synthetic chemical compounds in the olfactometer**. Bars indicate mean ±*SE*; means within rows followed by different alphabets indicate significant differences at *P* < 0.05. HC, healthy citrus; LC, Las-infected citrus; HCfP, healthy citrus infested with psyllids; LCfP, Las-infected citrus infested with psyllids.

**FIGURE 4:**
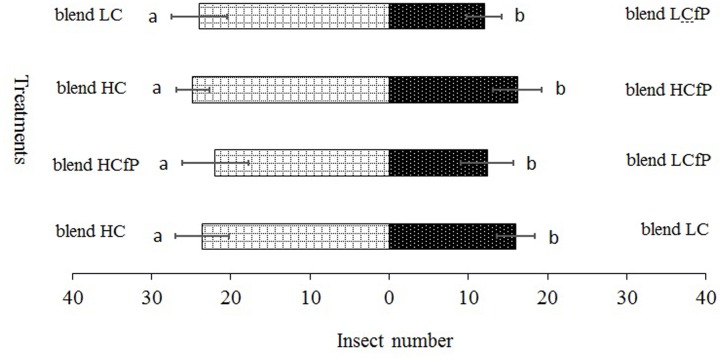
***Propylaea japonica* responses to the three different chemical compound blends in olfactometer**. (1) blend HC: D-limonene 24.75 nmol ml^-1^ and beta 1.00 nmol ml^-1^; (2) blend LC: D-limonene 17.32 nmol ml^-1^, methyl salicylate 0.06 nmol ml^-1^, and beta-ocimene 0.45 nmol ml^-1^; (3) blend HCfP: D-limonene 10.13 nmol ml^-1^, methyl salicylate 0.15 nmol ml^-1^, and beta-ocimene 0.20 nmol ml^-1^; (4) blend LCfP: D-limonene 1.06 nmol ml^-1^, methyl salicylate 0.18 nmol ml^-1^, and beta-ocimene 0.08 nmol ml^-1^. Bars indicate mean ±*SE*; means within rows followed by different alphabets indicate significant differences at *P* < 0.05.

Single dosages and blends of D-limonene, methyl salicylate, and beta-ocimene were used as odor sources for the olfactometer assays. The responses of *P. japonica* to the three synthetic compounds are shown in **Figures 3** and **4**.

In the single dosage treatments, D-limonene concentrations of 50, 25, 17, and 10 nmol ml^-1^ were significantly more attractive to *P. japonica* than fresh air (χ^2^ = 12.67, *df* = 4, *P* = 0.013; χ^2^ = 11.05, *df* = 4, *P* = 0.026; χ^2^ = 9.85, *df* = 4, *P* = 0.043; χ^2^ = 9.73, *df* = 4, *P* = 0.045, respectively). However, no significant differences were observed between fresh air and concentrations 0.1 nmol ml^-1^ (χ^2^ = 0.99, *df* = 4, *P* = 0.912) and 1 nmol ml^-1^ (χ^2^ = 1.00, *df* = 4, *P* = 0.910) (**Figure 3**). Similarly, in the single treatment of methyl salicylate, concentrations 0.64, 0.32, 0.18, and 0.15 nmol ml^-1^ had significantly high repellent effects on *P. japonica* compared to fresh air (χ^2^ = 10.45, *df* = 4, *P* = 0.033; χ^2^ = 10.45, *df* = 4, *P* = 0.033; χ^2^ = 9.95, *df* = 4, *P* = 0.041; χ^2^ = 11.35, *df* = 4, *P* = 0.023, respectively) while no significant differences were observed between fresh air and 0.03 nmol ml^-1^ (χ^2^ = 5.00, *df* = 4, *P* = 0.288) and 0.06 nmol ml^-1^ (χ^2^ = 2.51, *df* = 4, *P* = 0.644) concentrations (**Figure 3**). In the single treatment of beta-ocimene, concentrations 0.04, 0.08, and 0.2 nmol ml^-1^ showed significantly high attractive effect (χ^2^ = 11.78, *df* = 4, *P* = 0.019; χ^2^ = 9.69, *df* = 4, *P* = 0.046; χ^2^ = 11.53, *df* = 4, *P* = 0.021, respectively) to *P. japonica*, while 1.0 nmol ml^-1^ (χ^2^ = 13.91, *df* = 4, *P* = 0.008) and 2.0 nmol ml^-1^ (χ^2^ = 10.29, *df* = 4, *P* = 0.036) were significantly repellent to the predator compared to fresh air and the three remaining concentrations (0.04, 0.08, and 0.2 nmol ml^-1^) (**Figure 3**). However, there was no significant difference (χ^2^ = 1.51, *df* = 4, *P* = 0.825) between 0.45 nmol ml^-1^ and fresh air (**Figure 3**).

For the blend treatments, the results showed that the HC blend was more attractive to *P. japonica* than blends LC (χ^2^ = 12.20, *df* = 4, *P* = 0.016) and HCfP (χ^2^ = 10.06, *df* = 4, *P* = 0.039). Conversely, LCfP was less attractive to *P. japonica* than LC (χ^2^ = 11.29, *df* = 4, *P* = 0.024) and HCfP (χ^2^ = 13.44, *df* = 4, *P* = 0.009) (**Figure 4**).

## Discussion

Plants effectively use their secondary metabolites to protect themselves against herbivores and pathogens (Francis et al., 2004; Zhu and Park, 2005; Boone et al., 2008; Halitschke et al., 2008; Unsicker et al., 2015). At the same time, natural enemies use these induced compounds to seek their hosts. When insect vectors feed on pathogen infected plants, the defense mechanisms of the natural enemies, plants as well as the vectors are disturbed, due to changes in the synthesis of plant volatiles (Francis et al., 2004; Sullivan, 2005; Boone et al., 2008; Unsicker et al., 2015). In this study, we found that *D. citri*-infected citrus emitted less D-limonene and beta-ocimene, compared to citrus damaged by the vector insect as well as infected by Las; these plants produced high quantities of methyl salicylate. This finding suggested that the plant pathogen, Las, interacts with its vector *D. citri* and regulates volatiles emission from the host plant.

In the first olfactometer test using citrus with multiple treatments, we found that volatiles emitted from HC plants attracted the most *P. japonica*, while volatiles from citrus damaged by the vector as well as Las were less attractive to the vector. This result is inconsistent with previous findings from the olfactometer tests conducted in wasps (Gouinguene et al., 2003; D’Alessandro and Turlings, 2005; Dannon et al., 2010; Xu et al., 2014; Mutyambai et al., 2015; Takemoto and Takabayashi, 2015). It suggests that HC plants can produce volatiles that may attractant *P. japonica*, but these volatiles may be down-regulated by Las and psyllids through volatile profiles modification. In addition, our results also indicated that, the two pests (Las bacteria *Candidatus* Liberibacter asiaticus and *D. citri*) may induce citrus host plants to emit repellent chemicals for *P. japonica*. Simultaneous insect damage and pathogenic bacterial infection of citrus host plants have been reported to induce volatiles that modify not only the behavior of its insect vector (Mann et al., 2012) but also their natural enemies (Li et al., 2014). A number of studies have demonstrated that HIPVs emitted by infected plants attract more obligatory parasites or wasps (Francis et al., 2004; Sullivan, 2005; Zhu and Park, 2005; Boone et al., 2008; Halitschke et al., 2008; Zhang et al., 2009; Unsicker et al., 2015). The results of this study showed that infection of plants with pathogens through insect vectors may alter the balance and amounts of volatiles emitted by the infected plants thus reducing their attraction to a facultative predator, *P. japonica*.

Among the major compounds emitted from treated citrus plants, D-limonene, methyl salicylate and beta-ocimene had significant correlation with the olfactory responses of *P. japonica*, while others did not (Supplementary Table 3). In the bioassay responses of *P. japonica*, we found that synthetic D-limonene was attractive to *P. japonica* while synthetic methyl salicylate repelled it. Besides, synthetic beta-ocimene had dual effects by attracting the predator at lower concentrations and repelling it at high concentrations, regardless of whether the citrus plants were damaged by psyllids and/or Las. These three synthetic compounds were therefore found to have a significant impact on *P. japonica* behavior particularly in the search for prey on infested citrus plants. This result suggests that D-limonene and beta-ocimene may act as attractive cues, while methyl salicylate could serve as a repellent signal for *P. japonica*. D-limonene is one of most significant emitted volatiles of citrus and was previously identified to attract predatory beetles (Wheeler et al., 2002; Francis et al., 2004; Himanen et al., 2005; Zhu and Park, 2005; Heil and Silva Bueno, 2007; Wei et al., 2007; Boone et al., 2008; Halitschke et al., 2008; Zhang et al., 2009; Hare and Sun, 2011; Unsicker et al., 2015). Contrary to this, some studies also demonstrated that methyl salicylate could be used as an attractant cue for some beetle species (Francis et al., 2004; Boone et al., 2008; Halitschke et al., 2008; Zhang et al., 2009; Unsicker et al., 2015). Variations in the emitted quantities of these three major compounds may therefore explain the reduction in the attractiveness of Las-infected and/or psyllids-infested citrus host plants to *P. japonica*, compared to healthy plants. By comparing the three synthetic chemicals, D-limonene, beta-ocimene and methyl salicylate, we found that D-limonene is more attractive to *P. japonica* at concentrations 1nmol or less. On the contrary, methyl salicylate repelled the beetle, and the effect was more robust at higher concentrations. This suggests that the reduction in *P. japonica* attraction in treated citrus plants could have been caused by D-limonene (decreasing) and methyl salicylate (increasing). Interestingly, beta-ocimene showed both attractive and repellent activity at different concentrations and consequently, could be used together with D-limonene to increase *P. japonica* attraction in the management of *D. citri*.

It is interesting that psyllids induced volatiles from LC were less attractive to *P. japonica* than volatiles emitted from a HC plant since it is know that many insect infested host plants emit HIPVs to attract natural enemies, especially parasitoid wasps. One reason in the reduction of *P. japonica* prey performance might be due to changes in the physical constitution of psyllids; i.e., modifications caused by Las bacteria might not support the feeding and development of *P. japonica*. Since *P. japonica* is generalist ladybird beetle (Halitschke et al., 2008), it may exercise choice feeding on other prey and not feed on infected psyllids.

It seems that the vector and Las developed a strategy through long-term evolution to deceive infested citrus plants into repelling the ladybird beetles. This could be a mechanism by which herbivores fight their predators through avoidance behavior. It therefore suggests that the ladybird beetle, *P. japonica*, might not be a good biological candidate to be used to control vectors in some situations. On the other hand, some studies have shown that the volatiles from different cultivars, and the emission from resistant cultivar were more attractive to natural enemies than those from susceptible cultivars (Krips et al., 1996; Michereff et al., 2011). This may be due to the variation in volatile profiles therefore the combination of predator/prey/pathogens in different plant genetic backgrounds needs to be studied. Further studies are also warranted to understand why *P. japonica* reacts differently to the three emitted compounds. These findings may promote the application of volatiles and specifically D-limonene in biocontrol strategies.

## Author Contributions

Conceived and designed the experiments: YL, SL, and LW. Performed the experiments: YL and MH. Analyzed the data: YL, MH, and KA. Contributed reagents/materials/analysis tools: YL, SL, and KA. Wrote the paper: YL, MH, KA, and LW.

## Conflict of Interest Statement

The authors declare that the research was conducted in the absence of any commercial or financial relationships that could be construed as a potential conflict of interest.
