# Supplementary material for: Diaphorina citri Induces Huanglongbing-Infected Citrus Plant Volatiles to Repel and Reduce the Performance of Propylaea japonica
Source: Front Plant Sci. 2016 Dec 26;7:1969. doi: 10.3389/fpls.2016.01969 (PMC5183590; doi:10.3389/fpls.2016.01969)
Supplement: Supplementary file 1 [file Table_1.Pdf]

Insect Vector induces Huanglongbing-infected Plant Volatiles to repel and reduce the Performance of Predators

Supplementary Experiment 1: Detection for *Candidatus* Liberibacter asiaticus by running PCR

Extraction of total genomic DNA

Twenty microgram fresh leaf of sour orange and 10 *Diaphorina citri* were sampled to extract DNA. Genomic DNA of bacterial strains from citrus was extracted with a QIAprep® Spin Miniprep Kit according to the manufacturer's instructions.

PCR

Conventional PCR with TProfessional Standard Gradient Thermocycler (biomedizinische Analytik GmbH, Germany) was used to examine the presence of the HLB pathogen in the leaves of citrus plants collected from different treatments and *D. citri* collected from greenhouse. A forward primer: 5'-TGAATTCTTCGAGGTTGGTGAGC-3', reverse primer: 5'-AGAATTCGACTTAATCCCCACCT-3', designed on GenBank sequence M94319 of the HLB bacterium were used for PCR amplification in a 25-μl reaction volume. The PCR reaction consisted of 2 μl of DNA template, 1 μl of each 10-μM forward and reverse primer, 10μl 0.5-mM dNTPs, 2.5μl 10 × PCR buffer, and 0.25 μl (5 U/μl) of Go Taq Flexi DNA polymerase and 18.25μl ddH_2O and was amplified by using the following protocol: 94 ℃ for 2 min; followed by 35 cycles at 94 ℃ for 30 s, 54 ℃ for 20 s, and 72 ℃ for 60 s; followed by final extension at 72 ℃ for 10 min. PCR reaction (25 μl) was analyzed through 1.0% agarose gel in 1× Tris-acetate-EDTA buffer (40 mM Tris, 20 mM acetic acid, 1 mM EDTA, pH 8.5) and DNA bands were visualized by ethidium bromide staining (Supplementary Fig 1).

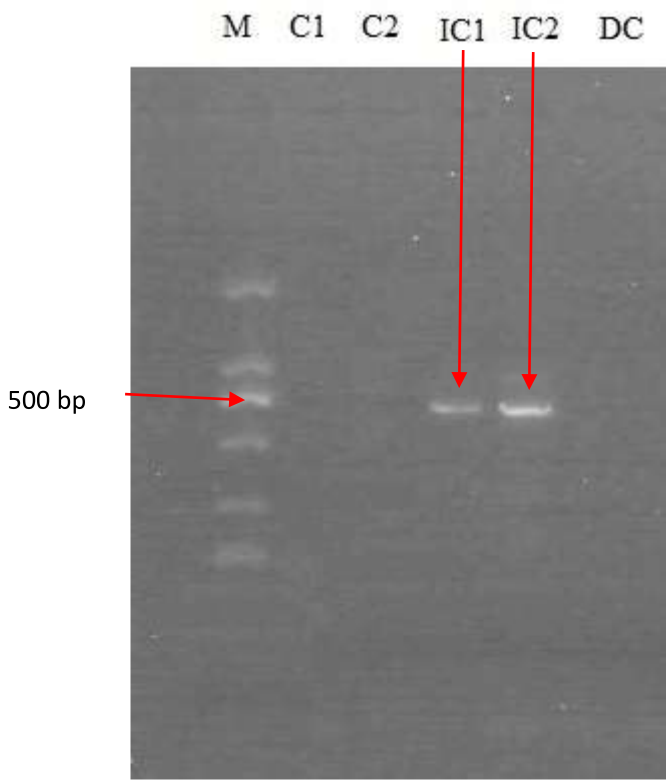

Supplementary Fig 1. Detection of *Candidatus* Liberibacter asiaticus infection with conventional polymerase chain reaction (PCR). Agarose gel electrophoresis of DNA amplified by SpeedSTAR HS DNA polymerase enzyme (Takara Bio USA) and Go Taq Flexi DNA polymerase enzyme (Promega Corp.) targeting the putative DNA polymerase gene sequence of *Candidatus* Liberibacter asiaticus. Total DNA was extracted from leaves of multiple treated sour orange and *D. citri*. M: DNA molecular weight size markers; C1: 12-month citrus seedlings used in the olfactory test; C2: six-month citrus seedlings used for Huanglongbing disease infection; IC1: Las-infected citrus plant; IC2: seedling plants from C2 kept in greenhouse for 6 months and DC: *D. citri* used in the experiment.

Supplementary Experiment 2: Olfactory analysis

The response of *P. japonica* to volatiles from different citrus plants were studied by two-choice tests. Y-tube olfactometer consisted of a 15 cm long stem and two 10 cm long arms, each with a 2 cm inner diameter was used in the tests. One single plant of healthy citrus (HC), Las-infected citrus (LC), healthy citrus infested with psyllids (HCfP) and Las-infected citrus infested with psyllids (LCfP) were individually put into the jar of headspace volatiles collecting instrument. Fresh air was blown or pumped into the jar with a pump from one side

at the rate of 300 ml·min^{-1}, and flowed out of the jar from the other side, and went into one arm of Y-tube at last. Fresh air was forced/ pumped into the other arm of Y-tube with a pump at the rate of 300 ml·min^{-1} directly (Fig 1).

Headspace detection

The key volatile compounds emitted from multi-treated citrus were detected for 12 h and identified by GC-MS, as described in the main text of this manuscript. The peak area of these main compounds in chromatogram was showed in Supplementary Table 2.

Correlation analysis

The peak area of the main volatile compounds and olfactory response of *P. japonica* to volatile blends emitted from multi-treated citrus were analyzed with Pearson correlation test. This test was used to distinguish the significant correlation between each compound and olfactory response (Supplementary Table 1).

Supplementary Table 1. Olfactory response of *Propylaea japonica* to volatile blends emitted from multi-treated citrus.

Treatments*	Mean ± STD**	95% confidence limited
HC	39.20 ± 2.86a	35.64 - 42.76
LC	32.20 ± 1.92b	29.81 - 34.59
HCfP	25.20 ± 2.86c	21.64 - 28.76
LCfP	18.40 ± 1.82d	16.14 - 20.66

* HC, healthy citrus; LC, Las-infected citrus; HCfP, healthy citrus fed by psyllids; LCfP, Las-infected citrus fed by psyllids. ** Means followed by the same letter in the column are not significantly different at $P < 0.05$ (Tukey's test, $P < 0.05$).

Supplementary Table 2. The peak area of the main volatile compounds emitted from multi-treated citrus.

Main compounds	Peak area × 10^{-6} pA s			
	HC*	LC	HCfP	LCfP
Benzaldehyde	7.16	37.16	46.77	12.12
1-Octen-3-ol	427.80	21.13	2.23	33.86
2-Methyl-6-heptene	289.42	292.29	287.50	308.29
Methyl jasmonate	3.54	160.96	107.53	159.78

D-Limonene	977.91	715.61	672.38	436.41
beta-Ocimene	484.06	200.98	288.67	274.66
Menthol	4.03	11.43	28.20	17.52
(E)-2-Nonenal	8.02	17.41	16.45	11.60
Terpineol	5.79	15.09	20.85	49.35
(E)-2-Hexena	13.85	16.39	5.06	32.69
3-Decanol	6.26	112.23	49.05	20.28
Methyl salicylate	6.67	267.66	1005.41	410.59
n-Hexyl acetate	11.99	6.97	5.49	5.38
(E)-2-Hexenol	71.98	8.44	14.36	30.38
n-Nonanal	54.49	52.69	7.58	14.19
n-Nonanol	21.14	21.73	9.53	26.89
(Z)-3-Hexenol	3.82	14.50	16.47	90.50

* HC, healthy citrus; LC, Las-infected citrus; HCfP, healthy citrus infested with psyllids; LCfP, Las-infected citrus infested with psyllids.

Supplementary Table 3. Correlation between olfactory response of *Propylaea japonica* to the multi-treated citrus plant volatiles and peak area of the main volatile compounds emitted from multi-treated citrus.

	Correlation coefficients between compounds and activity of volatile blends	
	Pearson Correlation	Sig. (2-tailed)
Benzaldehyde	-0.171	0.829
1-Octen-3-ol	0.760	0.240
2-Methyl-6-heptene	-0.701	0.299
Methyl jasmonate	-0.726	0.274
D-Limonene	0.970*	0.030
beta-Ocimene	0.951*	0.049
Menthol	-0.728	0.272
(E)-2-Nonenal	-0.294	0.706
Terpineol	-0.936	0.064
(E)-2-Hexena	-0.500	0.500
3-Decanol	0.054	0.946
Methyl salicylate	-0.956*	0.044
n-Hexyl acetate	0.888	0.112
(E)-2-Hexenol	0.540	0.460
n-Nonanal	0.866	0.134
n-Nonanol	0-.083	0.917
(Z)-3-Hexenol	-0.845	0.155

*Correlation coefficients with significant differences.

Supplementary Experiment 3

In this pre-experiment, three chemicals, D-limonene (DL), methyl salicylate (MeSA) and beta-ocimene (BO) were applied on the cotton roll at different quantities of 1, 10, 100 and 1000 nmol (chemicals were dissolved and diluted with TEC), respectively. The cotton roll was then put into the jar of headspace collecting instrument, and Tanax A was used as absorbent in this experiment. Clean air was forced/ blown into the jar at the rate of 300 ml min^{-1} with the pump. GC-MS was used to detect the quantities with the same plotting parameters as described above.

The emission rate of D-limonene, MeSA and beta-ocimene were showed in Supplementary Table 4. According to the results of GC-MS in Table 1 and regression equation of Supplementary Table 4, the selected concentrations of the synthetic chemical of D-limonene, MeSA and beta-ocimene applied in the experiment are shown in Supplementary Tables5.

Supplementary Table 4. Quantities of the key chemicals emitted from the cotton roll in different treatments in 12 hours

Chemicals*	Quantities of chemicals emitted from cotton roll (nmol) ± SE						Regression equation
	0.05	0.1	1	10	25	50	
DL	0.04±0.01	0.08±0.01	0.81±0.02	7.27±0.42	14.39±1.45	26.50±3.65	$y=-0.005x^2+0.66x$
MeSA	0.04±0.01	0.08±0.01	0.73±0.01	4.63±0.12	7.81±0.17	14.33±0.71	$y=-0.0038x^2+0.52x$
BO	0.04±0.01	0.08±0.01	0.72±0.04	6.13±0.23	11.23±1.21	19.17±2.71	$y=-0.005x^2+0.62x$

*DL, D-limonene; MeSA, methyl salicylate; BO, beta-ocimene

Supplementary Table 5. Predicted concentrations of synthetic compounds applied in the experiment.

	Quantities detected in plant volatiles by GC-MS*			Predicted concentrations of synthetic compounds		
	DL	MeSA	BO	DL	MeSA	BO
HC**	13.45	ND	0.62	24.75	0	1.01
LC	10	0.028	0.28	17.32	0.06	0.45
HCfP	6.26	0.055	0.12	10.13	0.15	0.20
LCfP	0.70	0.097	0.051	1.06	0.18	0.08

*Data were obtained from Table 1.

** HC, healthy citrus; LC, Las-infected citrus; HCfP, healthy citrus infested with psyllids; LCfP, Las-infected citrus infested with psyllids. DL, D-limonene; MeSA, methyl salicylate and BO, beta-ocimene.
